# Synergistic impact of psoriasis and hypertension on all-cause mortality risk: A prospective cohort study

**DOI:** 10.1371/journal.pone.0306048

**Published:** 2024-07-05

**Authors:** Honglei Zhao, Ji Wu, Qianqian Wu

**Affiliations:** Department of Dermatology, Beilun District People’s Hospital, Ningbo, China; Sukh Sagar Medical College and Hospital, INDIA

## Abstract

**Background:**

The linkage between psoriasis and hypertension has been established through observational studies. Despite this, a comprehensive assessment of the combined effects of psoriasis and hypertension on all-cause mortality is lacking. The principal aim of the present study is to elucidate the synergistic impact of psoriasis and hypertension on mortality within a representative cohort of adults residing in the United States.

**Methods:**

The analysis was conducted on comprehensive datasets derived from the National Health and Nutrition Examination Study spanning two distinct periods: 2003–2006 and 2009–2014. The determination of psoriasis status relied on self-reported questionnaire data, whereas hypertension was characterized by parameters including systolic blood pressure ≥ 140 mmHg, diastolic blood pressure ≥ 90 mmHg, self-reported physician diagnosis, or the use of antihypertensive medication. The assessment of the interplay between psoriasis and hypertension employed multivariable logistic regression analyses. Continuous monitoring of participants’ vital status was conducted until December 31, 2019. A four-level variable amalgamating information on psoriasis and hypertension was established, and the evaluation of survival probability utilized the Kaplan-Meier curve alongside Cox regression analysis. Hazard ratios (HRs) and their associated 95% confidence intervals (CIs) were computed to scrutinize the correlation between psoriasis/hypertension and all-cause mortality.

**Results:**

In total, this study included 19,799 participants, among whom 554 had psoriasis and 7,692 had hypertension. The findings from the logistic regression analyses indicated a heightened risk of hypertension among individuals with psoriasis in comparison to those devoid of psoriasis. Throughout a median follow-up spanning 105 months, 1,845 participants experienced all-cause death. In comparison to individuals devoid of both hypertension and psoriasis, those with psoriasis alone exhibited an all-cause mortality HR of 0.73 (95% CI: 0.35–1.53), individuals with hypertension alone showed an HR of 1.78 (95% CI: 1.55–2.04), and those with both psoriasis and hypertension had an HR of 2.33 (95% CI: 1.60–3.40). In the course of a stratified analysis differentiating between the presence and absence of psoriasis, it was noted that hypertension correlated with an elevated risk of all-cause mortality in individuals lacking psoriasis (HR 1.77, 95% CI: 1.54–2.04). Notably, this association was further accentuated among individuals with psoriasis, revealing an increased HR of 3.23 (95% CI: 1.47–7.13).

**Conclusions:**

The outcomes of our investigation demonstrated a noteworthy and positive association between psoriasis, hypertension, and all-cause mortality. These findings indicate that individuals who have both psoriasis and hypertension face an increased likelihood of mortality.

## Introduction

Hypertension, a widely prevalent chronic condition, manifests its impact on a substantial proportion of the worldwide population. Data provided by the World Health Organization indicates an estimated prevalence of approximately 40% among adults and 4% among individuals aged 19 and below on a global scale. Furthermore, its prevalence is on the rise, making it a notable risk determinant contributing substantially to the overarching burden of global morbidity [1–4]. Extended durations of heightened blood pressure are conducive to vascular endothelial dysfunction and the enduring activation of inflammatory mediators. Consequently, hypertension exhibits a notable correlation with cardiovascular disease and is acknowledged as the primary contributor to both cardiovascular ailments and premature mortality on a global scale [3].

Psoriasis, referred to as chronic plaque or psoriasis vulgaris, represents an enduring inflammatory dermatological condition that exerts its influence on a substantial segment of the adult demographic within the United States, affecting around 3.0% or over 7.5 million adults. This condition imposes a significant socioeconomic burden on individuals and society as a whole [5, 6]. Psoriasis represents an immune-mediated inflammatory disorder of the skin marked by a propensity for recurring episodes over temporal intervals [7]. Recent evidence indicates that psoriasis is not solely limited to skin manifestations but rather a systemic inflammatory disease that is linked to various comorbidities. The array of comorbidities, including kidney disease, metabolic syndrome, and cardiovascular disorders, accentuates the multisystemic nature of psoriasis, underscoring its intricate association with inflammatory processes manifesting across the systemic landscape of the human body [8–10].

Through a comprehensive systematic review and meta-analysis of observational studies, a definitive association between psoriasis and hypertension has been established [11, 12]. In a meta-analysis by Duan et al., odds ratios (ORs) adjusted for covariates were employed, revealing an increased likelihood of developing hypertension in individuals with psoriasis in comparison to their psoriasis-free counterparts [11]. Furthermore, there seems to be a correlation between the severity of psoriasis and an augmented susceptibility to hypertension, with patients experiencing more severe symptoms having greater odds of developing hypertension [12]. Importantly, psoriasis has been delineated as a distinct risk factor for mortality based on findings from a cohort study carried out in the United States [13]. Despite this, the collective influence of psoriasis and hypertension on all-cause mortality has not been thoroughly explored up to this point.

Given the significant prevalence of hypertension within the population [1–4], it is imperative to investigate comprehensively the collective influence of psoriasis and hypertension on mortality risk. Utilizing datasets derived from the National Health and Nutrition Examination Survey (NHANES) spanning the years 2003–2006 and 2009–2014, this investigation aimed to scrutinize the intricate relationship among psoriasis, hypertension, and all-cause mortality. The principal conjecture postulated that individuals presenting with either psoriasis or hypertension would exhibit a heightened mortality rate relative to those lacking both conditions, with the most pronounced risk discerned among individuals concurrently experiencing psoriasis and hypertension.

## Materials and methods

### Study population and design

As a tool, the NHANES program holds significance as a valuable cross-sectional means for systematically gauging the overall health and nutritional status of the American population. It utilizes a combination of interviews and physical examinations to collect comprehensive data on demographics, dietary habits, medical examinations, laboratory results, and questionnaire responses. Approval for the NHANES protocol has been granted by the ethics review board of the National Center for Health Statistics. All individuals participating in the program provide written informed consent, safeguarding both their voluntary participation and rights. Given that the present study constitutes a secondary analysis of publicly available NHANES data, no institutional review board approval was required or sought.

In the current investigation, extensive data from five consecutive NHANES cycles were employed, specifically from the years 2003–2004, 2005–2006, 2009–2010, 2011–2012 and 2013–2014. The study cohort was composed of adult individuals who underwent a dual-phase process, initiating with an initial residence-based interview to elicit sociodemographic information and medical history, succeeded by a thorough assessment at a mobile examination center. In order to uphold the quality and integrity of the analysis, we selectively included adult participants who possessed complete data on both psoriasis and hypertension. Consequently, individuals with missing information on covariates were excluded from the analytical population.

### Diagnostic of psoriasis and hypertension

To ascertain the presence of psoriasis among participants, data collection relied on self-administered questionnaires during the 2003–2006 and 2009–2014 NHANES surveys. For the identification of individuals with psoriasis, an affirmative response to the query, “Have you ever been told by a health care provider that you had psoriasis?” was regarded as indicative of the condition. The NHANES survey provided questions about the psoriasis status during the periods 2003–2016 and 2009–2014. Thus, we could only select these periods for data collection and analysis.

Individuals were categorized as having hypertension if they manifested a systolic blood pressure of 140 mmHg or above, or a diastolic blood pressure of 90 mmHg or above. Furthermore, a self-reported diagnosis of hypertension by a physician or the utilization of antihypertensive medication were also regarded as markers of the condition.

### Covariates

Control variables, encompassing potential confounding factors in this study, comprised socioeconomic and health-related elements. Socioeconomic attributes comprised sex, age, race, education level, and marital status. Health-related characteristics encompassed body mass index (BMI), smoking and drinking status. The classification of smoking status was delineated into three distinct groups: those identified as never smokers (having consumed less than 100 cigarettes in their lifetime), former smokers (having smoked more than 100 cigarettes in their lifetime and currently abstaining from smoking), and current smokers (having smoked more than 100 cigarettes in their lifetime and currently engaging in smoking either intermittently or daily). The determination of diabetes was characterized by criteria encompassing a diagnosed status of diabetes, current use of glucose-lowering medication, or meeting defined benchmarks including HbA1c ≥ 6.5%, fasting serum glucose level ≥ 7.0 mmol/L, or random blood glucose ≥ 11.1 mmol/L.

### Ascertainment of mortality

The NCHS database provides NHANES public-use linked mortality information up to December 31, 2019. These datasets were cross-referenced with the National Death Index to determine the survival status of participants. For the validation of survival status, a set of 12 distinct characteristics were acquired to establish linkage between NHANES samples and the National Death Index. For surviving participants, the calculation of survival time (expressed in months) was derived from the commencement of the initial interview to either the date of mortality or the termination of the follow-up period (December 31, 2019). The primary objective of our analysis was to investigate all-cause mortality as the designated outcome variable.

### Statistical analysis

The comprehensive and intricate sampling design adopted in the NHANES involved a complex multistage, stratified, cluster sampling methodology. During the subsequent data analysis phase, appropriate weighting procedures were executed to generate nationally representative estimates [14]. The analytical processes encompassed a thorough consideration of the intricate sample design, where mobile examination center sample weights were systematically applied for the computation of estimates. Baseline demographic and clinical characteristics were succinctly summarized and systematically stratified based on the status of psoriasis and hypertension. Survey-weighted means with 95% confidence intervals (CIs) were utilized to report continuous variables, whereas survey-weighted percentages with 95% CI were employed for the presentation of dichotomous variables. The comparison of continuous variables between different groups was executed using weighted t-tests, while the examination of categorical variables involved the application of chi-square tests, both adjusted for survey weights.

A multivariate logistic regression model was applied to compute ORs and their corresponding 95% CIs for the examination of the association between psoriasis and hypertension. This analysis involved the adjustment for social demographic covariates. Additionally, survival rates across distinct groups were evaluated using the Kaplan-Meier method. Subsequently, univariate and multivariate Cox proportional-hazards regression models were deployed to compute hazard ratios (HRs) along with their respective 95% CIs, aiming to scrutinize the association between psoriasis and all-cause mortality, taking hypertension status into account. For the evaluation of combined effects, participants were classified based on the binary presence of psoriasis and hypertension. The comparison of HRs for mortality was conducted across four categories: individuals without psoriasis or hypertension, those with only psoriasis, those with only hypertension, and those with both psoriasis and hypertension. Model 1 reflected the unadjusted crude model, and Model 2 entailed adjustments for sex, age, race, education level, marital status, and BMI category. Additionally, Model 3 introduced further adjustments for smoking status, drinking status, and diabetes.

The analytical procedures were conducted utilizing R software, specifically version 4.2.2. Statistical significance was assessed with a two-sided *P*-value < 0.05 serving as the predetermined threshold.

## Results

**Fig 1:**
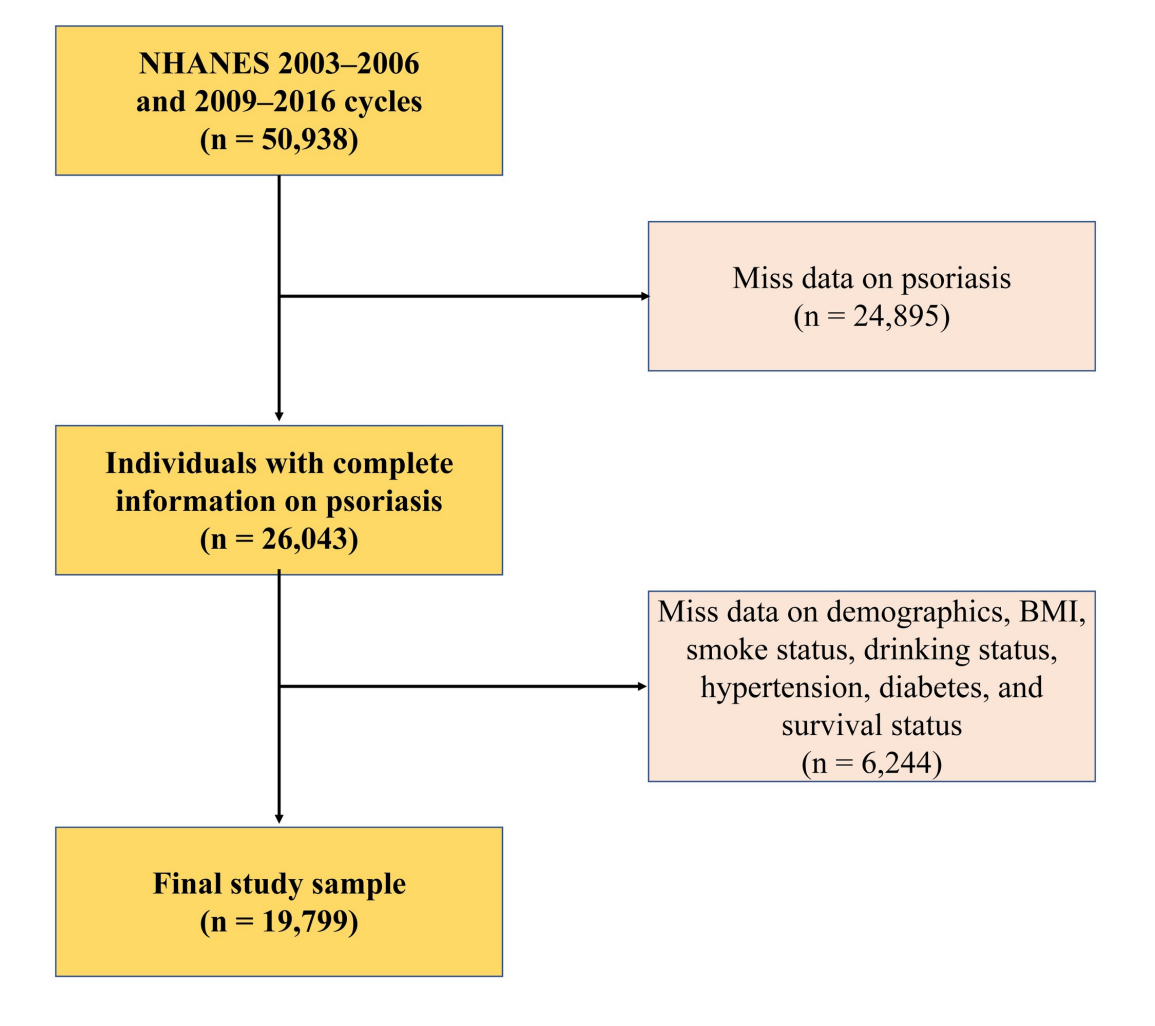
Flowchart of the study population.

The inclusion and exclusion criteria for the present study are graphically illustrated in Fig 1. A total of 19,799 participants, as specified in Fig 1, were enrolled in the study. Table 1 provides an exposition of the demographic and clinical characteristics of participants, both in aggregate and segregated by their respective psoriasis and hypertension status. Within the study population, the occurrence of psoriasis was observed in 2.798%, and hypertension was prevalent in 38.85%. Notably, among the 7,692 individuals diagnosed with hypertension, 276 had a historical record of psoriasis. The mean age of the participants was 45 years, with 49.91% being male and 69.01% identifying as non-Hispanic white. Within the subset of individuals with hypertension, those concurrently affected by psoriasis exhibited a higher likelihood of being female, non-Hispanic white, and former smokers. Correspondingly, in the subset without hypertension, individuals with psoriasis were also more inclined to be non-Hispanic white and former smokers.

**Table 1:** Baseline characteristics of study population by presence of hypertension and psoriasis.

		Hypertension			No Hypertension		
Variable	Overall (N = 19799)	No Psoriasis (N = 7416)	Psoriasis (N = 276)	*P*-value	No Psoriasis (N = 11829)	Psoriasis (N = 278)	*P*-value
Age (years)	45.04 (44.49, 45.60)	54.20 (53.59, 54.81)	55.30 (53.14, 57.45)	0.31	40.07 (39.59, 40.55)	41.55 (39.98, 43.12)	0.06
Gender, n (%)				0.07			0.67
Female	50.09 (47.31, 52.86)	48.98 (47.73, 50.22)	55.94 (48.48, 63.40)		50.56 (49.63, 51.49)	49.25 (43.36, 55.13)	
Male	49.91 (47.11, 52.72)	51.02 (49.78, 52.27)	44.06 (36.60, 51.52)		49.44 (48.51, 50.37)	50.75 (44.87, 56.64)	
Race				< 0.0001			< 0.0001
Hispanic	5.03 (4.06, 6.00)	3.99 (2.94, 5.04)	2.86 (0.95, 4.77)		5.64 (4.54, 6.74)	4.72 (2.63, 6.80)	
Non-Hispanic White	69.01 (62.61, 75.41)	70.05 (66.63, 73.47)	83.75 (79.02, 88.48)		67.85 (64.90, 70.80)	79.71 (75.46, 83.96)	
Non-Hispanic Black	11.12 (9.92, 12.33)	14.35 (12.18, 16.52)	6.26 (3.90, 8.63)		9.68 (8.40, 10.96)	5.39 (3.30, 7.49)	
Mexican American	8.36 (6.97, 9.75)	5.93 (4.41, 7.45)	2.88 (1.09, 4.67)		9.87 (8.16, 11.59)	4.41 (1.99, 6.84)	
Other	6.47 (5.76, 7.19)	5.68 (4.76, 6.60)	4.25 (1.58, 6.92)		6.96 (6.18, 7.74)	5.77 (3.36, 8.17)	
Education				0.39			0.07
Less than high school	15.67 (14.33, 17.02)	18.03 (16.51, 19.55)	15.34 (10.49, 20.19)		14.57 (13.26, 15.87)	10.74 (6.74, 14.75)	
High school or equivalent	22.78 (20.88, 24.67)	24.64 (23.01, 26.28)	24.68 (18.01, 31.34)		21.88 (20.62, 23.14)	17.95 (13.24, 22.66)	
Some college or AA degree	32.77 (30.84, 34.70)	32.89 (31.53, 34.24)	38.31 (30.95, 45.66)		32.58 (31.09, 34.06)	32.96 (25.99, 39.93)	
College graduate or above	28.78 (26.21, 31.35)	24.44 (22.31, 26.56)	21.68 (16.01, 27.34)		30.98 (28.85, 33.11)	38.35 (30.53, 46.16)	
Marital status				0.84			0.99
Married	55.67 (51.65, 59.69)	58.81 (56.97, 60.66)	60.79 (53.90, 67.68)		53.95 (52.17, 55.72)	53.70 (46.60, 60.81)	
Never married	19.12 (17.88, 20.37)	11.22 (10.14, 12.30)	9.00 (4.56, 13.44)		23.42 (21.66, 25.19)	23.01 (16.84, 29.18)	
Living with partner	7.99 (7.27, 8.71)	5.77 (5.10, 6.44)	5.81 (2.42, 9.21)		9.16 (8.46, 9.86)	9.73 (5.43, 14.04)	
Other	17.22 (16.05, 18.39)	24.20 (23.00, 25.40)	24.39 (17.45, 31.34)		13.47 (12.66, 14.27)	13.55 (9.56, 17.55)	
BMI category				0.06			0.17
<25	31.37 (29.35, 33.40)	18.89 (17.86, 19.91)	12.56 (8.43, 16.68)		38.37 (36.74, 39.99)	32.21 (25.80, 38.62)	
25–30	32.86 (30.72, 35.01)	31.97 (30.65, 33.29)	37.23 (29.65, 44.80)		33.21 (31.87, 34.55)	33.80 (27.04, 40.56)	
> = 30	35.77 (33.51, 38.02)	49.14 (47.65, 50.63)	50.21 (42.92, 57.51)		28.42 (27.18, 29.66)	33.99 (26.48, 41.51)	
Smoking status				< 0.0001			0.02
never	54.24 (51.36, 57.12)	50.35 (48.64, 52.05)	36.19 (29.90, 42.47)		56.82 (55.27, 58.38)	49.52 (42.41, 56.64)	
former	23.12 (21.11, 25.13)	29.27 (27.75, 30.79)	45.76 (38.26, 53.26)		19.27 (17.96, 20.58)	26.87 (21.17, 32.56)	
now	22.64 (21.09, 24.19)	20.39 (18.98, 21.80)	18.05 (13.43, 22.67)		23.91 (22.56, 25.25)	23.61 (17.76, 29.46)	
Drinking status				0.22			0.59
former	10.40 (9.17, 11.62)	11.28 (10.07, 12.48)	8.05 (5.27, 10.82)		10.01 (8.74, 11.29)	9.20 (5.04, 13.36)	
never	14.55 (13.15, 15.96)	19.69 (18.25, 21.13)	19.05 (13.47, 24.63)		11.76 (10.73, 12.79)	13.93 (8.93, 18.93)	
now	75.05 (70.78, 79.32)	69.03 (67.12, 70.95)	72.90 (66.54, 79.26)		78.23 (76.34, 80.12)	76.87 (71.03, 82.72)	
Diabetes				0.4			0.66
no	81.58 (76.99, 86.17)	66.69 (65.52, 67.86)	70.80 (64.17, 77.43)		89.53 (88.68, 90.39)	87.54 (83.43, 91.64)	
IGT	3.33 (2.95, 3.72)	4.79 (4.22, 5.35)	2.81 (0.81, 4.80)		2.59 (2.17, 3.01)	2.87 (0.80, 4.95)	
IFG	3.22 (2.86, 3.57)	5.09 (4.30, 5.87)	3.77 (1.03, 6.50)		2.21 (1.86, 2.57)	3.33 (0.55, 6.11)	
DM	11.87 (11.00, 12.73)	23.44 (22.28, 24.60)	22.63 (17.02, 28.24)		5.66 (5.06, 6.27)	6.26 (3.66, 8.86)	

Values for continuous variables, as survey-weighted mean (95% CI), P-value was calculated by survey-weighted linear regression. Values for categorical variables are given as survey-weighted, percentage (95% CI), P-value was calculated by survey-weighted Chi-square test.

BMI, body mass index; DM, diabetes mellitus; IFG, impaired fasting glycaemia; IGT, impaired glucose tolerance.

Table 2 reveals the outcomes of logistic regression analyses, elucidating a positive correlation between psoriasis and the incidence of hypertension. Across Model 1 (OR = 1.60; 95% CI: 1.28–2.01), Model 2 (OR = 1.47; 95% CI: 1.13–1.92), and Model 3 (OR = 1.47; 95% CI: 1.13–1.90), individuals with psoriasis consistently demonstrated an elevated risk of hypertension compared to their counterparts without psoriasis. A comprehensive multivariate regression analysis further identified significant associations of sex, age, race, education level, marital status, BMI, and smoking status with hypertension. Moreover, diabetes (OR = 2.53; 95% CI: 2.19–2.91) emerged as a significant factor significantly linked to an increased risk of hypertension.

**Table 2:** Relationship between psoriasis and hypertension among adults aged 20 years or older.

Variable	Model 1		Model 2		Model 3	
	OR (95% CI)	*P*-value	OR (95% CI)	*P*-value	OR (95% CI)	*P*-value
Psoriasis						
No	ref	ref	ref	ref	ref	ref
Yes	1.60 (1.28, 2.01)	<0.0001	1.47 (1.13, 1.92)	0.005	1.47 (1.13, 1.90)	0.005
Sex						
Female			ref	ref	ref	ref
Male			1.20 (1.11, 1.30)	<0.0001	1.14 (1.05, 1.24)	0.002
Age group						
≤49			ref	ref	ref	ref
50–65			3.45 (3.12, 3.82)	<0.0001	3.03 (2.73, 3.37)	<0.0001
≥65			9.08 (7.98, 10.33)	<0.0001	7.27 (6.28, 8.41)	<0.0001
Race						
Hispanic			ref	ref	ref	ref
Non-Hispanic White			1.30 (1.07, 1.58)	0.01	1.35 (1.11, 1.64)	0.003
Non-Hispanic Black			2.11 (1.73, 2.57)	<0.0001	2.18 (1.80, 2.63)	<0.0001
Mexican American			0.83 (0.67, 1.03)	0.09	0.83 (0.67, 1.02)	0.08
Other			1.53 (1.19, 1.97)	0.001	1.48 (1.15, 1.91)	0.003
Education						
Less than high school			ref	ref	ref	ref
High school or equivalent			0.88 (0.77, 1.00)	0.05	0.93 (0.81, 1.06)	0.25
Some college or AA degree			0.83 (0.73, 0.94)	0.003	0.88 (0.78, 1.00)	0.05
College graduate or above			0.64 (0.56, 0.74)	<0.0001	0.72 (0.62, 0.83)	<0.0001
Marital status						
Married			ref	ref	ref	ref
Never married			0.69 (0.62, 0.77)	<0.0001	0.71 (0.63, 0.79)	<0.0001
Living with partner			0.88 (0.75, 1.03)	0.11	0.88 (0.76, 1.04)	0.13
Other			1.21 (1.09, 1.34)	<0.001	1.18 (1.06, 1.32)	0.003
BMI category						
<25			ref	ref	ref	ref
25–30			1.80 (1.62, 2.00)	<0.0001	1.73 (1.56, 1.92)	<0.0001
≥30			3.50 (3.18, 3.85)	<0.0001	3.00 (2.73, 3.30)	<0.0001
Smoking status						
Never					ref	ref
Former					1.17 (1.03, 1.32)	0.02
Now					1.12 (1.00, 1.27)	0.05
Drinking status						
Never					ref	ref
Former					1.12 (0.94, 1.33)	0.2
Now					1.02 (0.89, 1.18)	0.76
Diabetes						
No					ref	ref
IGT					1.64 (1.25, 2.16)	<0.001
IFG					2.00 (1.54, 2.60)	<0.0001
DM					2.53 (2.19, 2.91)	<0.0001

Model 1, unadjusted crude model.

Model 2, adjusted for demographic characteristics (sex, age group, race, education, marital status); BMI category

Model 3, adjusted for demographic characteristics (sex, age group, race, education, marital status); BMI category, smoking status, drinking status and diabetes.

BMI, body mass index; CI, confidence interval; DM, diabetes mellitus; IFG, impaired fasting glycaemia; IGT, impaired glucose tolerance; OR, odds ratio.

Across a median follow-up duration of 105 months, 1,845 participants succumbed to all-cause mortality. Multivariable analyses indicated that individuals with psoriasis were at a 1.17-fold higher risk of increased all-cause mortality (95% CI: 0.84–1.62). Furthermore, a statistically significant association was established between hypertension and all-cause mortality (HR 1.81, 95% CI: 1.58–2.07) (S1 Table). Moreover, significant associations with all-cause mortality were discerned for sex, age, race, education level, marital status, BMI, smoking status, drinking status, and diabetes (S1 Table).

**Fig 2:**
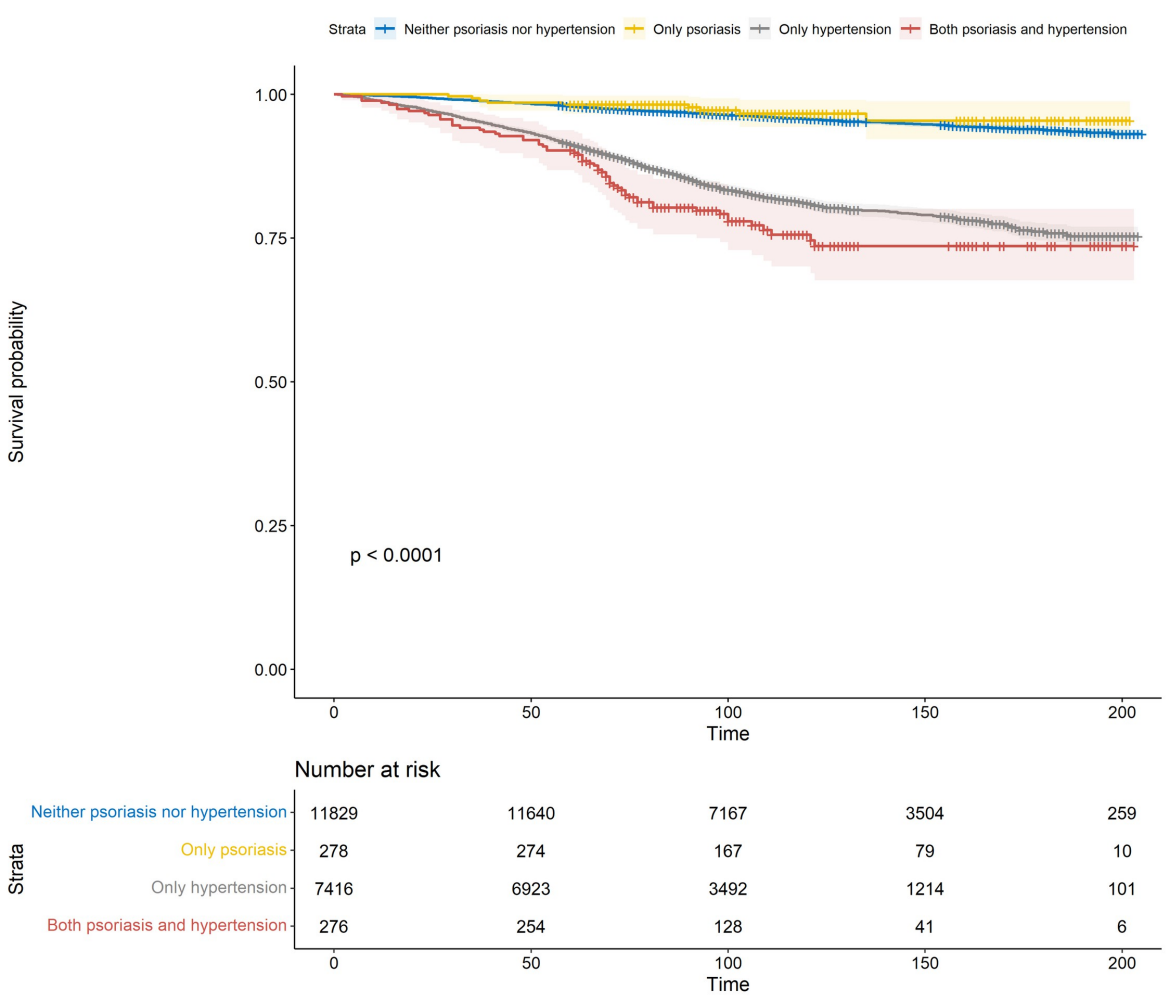
Association between psoriasis and all-cause mortality based on hypertension status.

To assess the influence of hypertension and/or psoriasis on all-cause mortality, participants were categorized into four cohorts: (i) individuals devoid of both hypertension and psoriasis; (ii) those presenting with psoriasis but devoid of hypertension; (iii) individuals with hypertension but lacking psoriasis; and (iv) those concurrently affected by both hypertension and psoriasis. Time-to-event analyses were employed to investigate the impact of psoriasis and hypertension on all-cause mortality. The Kaplan-Meier survival curves in Fig 2 revealed a noteworthy difference (log-rank, *P* < 0.0001) in all-cause mortality across the four groups, with the highest mortality rate observed in participants with both psoriasis and hypertension.

The findings from the weighted Cox proportional hazards regression analysis are presented in Table 3. In the unadjusted assessment, individuals with both psoriasis and hypertension displayed an approximately 5.53-fold heightened risk of all-cause mortality compared to the reference group (*P* < 0.0001). This elevated risk persisted following adjustments for demographic factors (Model 2, *P* < 0.0001) and in the fully adjusted model (Model 3, *P* < 0.0001). Model 3 revealed, in comparison to individuals without hypertension and psoriasis, HRs for all-cause mortality of 0.73 (95% CI: 0.35–1.53) for those with psoriasis only, 1.78 (95% CI: 1.55–2.04) for individuals with hypertension only, and 2.33 (95% CI: 1.60–3.40) for individuals with both psoriasis and hypertension.

**Table 3:** Association between psoriasis/hypertension status and all-cause mortality.

Variable	Model 1		Model 2		Model 3	
	HR (95% CI)	*P*-value	HR (95% CI)	*P*-value	HR (95% CI)	*P*-value
Group						
Neither psoriasis nor hypertension	ref	ref	ref	ref	ref	ref
Only psoriasis	0.74 (0.37, 1.52)	0.42	0.79 (0.37, 1.65)	0.52	0.73 (0.35, 1.53)	0.41
Only hypertension	4.27 (3.80, 4.80)	<0.0001	1.90 (1.66, 2.17)	<0.0001	1.78 (1.55, 2.04)	<0.0001
Both psoriasis and hypertension	5.53 (4.05, 7.57)	<0.0001	2.36 (1.67, 3.34)	<0.0001	2.33 (1.60, 3.40)	<0.0001
Sex						
Female			ref	ref	ref	ref
Male			1.51 (1.37, 1.67)	<0.0001	1.41 (1.28, 1.56)	<0.0001
Age group						
≤49			ref	ref	ref	ref
50–65			3.76 (3.03, 4.66)	<0.0001	3.38 (2.71, 4.21)	<0.0001
≥65			14.86 (11.81,18.70)	<0.0001	13.28 (10.53,16.75)	<0.0001
Race						
Hispanic			ref	ref	ref	ref
Non-Hispanic White			1.74 (1.28, 2.37)	<0.001	1.70 (1.26, 2.27)	<0.001
Non-Hispanic Black			1.64 (1.21, 2.21)	0.001	1.49 (1.11, 2.00)	0.01
Mexican American			1.14 (0.86, 1.52)	0.37	1.12 (0.85, 1.49)	0.42
Other			1.30 (0.89, 1.89)	0.17	1.15 (0.78, 1.71)	0.48
Education						
Less than high school			ref	ref	ref	ref
High school or equivalent			0.74 (0.60, 0.92)	0.01	0.84 (0.68, 1.03)	0.09
Some college or AA degree			0.62 (0.50, 0.76)	<0.0001	0.74 (0.60, 0.90)	0.003
College graduate or above			0.42 (0.35, 0.52)	<0.0001	0.59 (0.48, 0.72)	<0.0001
Marital status						
Married			ref	ref	ref	ref
Never married			1.72 (1.37, 2.14)	<0.0001	1.70 (1.35, 2.14)	<0.0001
Living with partner			1.76 (1.31, 2.38)	<0.001	1.56 (1.15, 2.12)	0.004
Other			1.83 (1.59, 2.11)	<0.0001	1.73 (1.50, 2.00)	<0.0001
BMI category						
<25			ref	ref	ref	ref
25–30			0.75 (0.65, 0.88)	<0.001	0.77 (0.67, 0.89)	<0.001
≥30			0.87 (0.75, 1.00)	0.04	0.81 (0.71, 0.93)	0.003
Smoking status						
Never					ref	ref
Former					1.19 (1.02, 1.40)	0.03
Now					2.01 (1.73, 2.32)	<0.0001
Drinking status						
Never					ref	ref
Former					1.34 (1.09, 1.65)	0.01
Now					0.77 (0.62, 0.96)	0.02
Diabetes						
No					ref	ref
IGT					1.22 (0.92, 1.60)	0.16
IFG					1.33 (1.00, 1.77)	0.05
DM					1.70 (1.47, 1.98)	<0.0001

Model 1, unadjusted crude model.

Model 2, adjusted for demographic characteristics (sex, age group, race, education, marital status); BMI category

Model 3, adjusted for demographic characteristics (sex, age group, race, education, marital status); BMI category, smoking status, drinking status and diabetes.

BMI, body mass index; CI, confidence interval; DM, diabetes mellitus; HR, hazard ratio; IFG, impaired fasting glycaemia; IGT, impaired glucose tolerance.

Conducting a stratified analysis based on the presence or absence of psoriasis (Table 4), we identified a correlation between hypertension and an elevated risk of all-cause mortality among individuals lacking psoriasis (HR 1.77, 95% CI: 1.54–2.04). Interestingly, this association was notably intensified in individuals with psoriasis (HR 3.23, 95% CI: 1.47–7.13).

**Table 4:** Association between psoriasis and all-cause mortality stratified by presence or absence of hypertension.

Variable	No psoriasis		Psoriasis	
	HR (95% CI)	*P*-value	HR (95% CI)	*P*-value
Hypertension				
No	ref	ref	ref	ref
Yes	1.77 (1.54, 2.04)	<0.0001	3.23 (1.47, 7.13)	0.004
Sex				
Female	ref	ref	ref	ref
Male	1.43 (1.28, 1.59)	<0.0001	1.16 (0.62, 2.19)	0.64
Age group				
≤49	ref	ref	ref	ref
50–65	3.26 (2.61, 4.07)	<0.0001	11.04 (4.44, 27.44)	<0.0001
≥65	13.29 (10.50, 16.81)	<0.0001	28.22 (7.65, 104.13)	<0.0001
Race				
Hispanic	ref	ref	ref	ref
Non-Hispanic White	1.68 (1.26, 2.25)	<0.001	5.56 (1.41, 21.93)	0.01
Non-Hispanic Black	1.49 (1.11, 2.01)	0.01	2.33 (0.46, 11.78)	0.31
Mexican American	1.09 (0.83, 1.45)	0.53	1.36 (0.16, 11.78)	0.78
Other	1.10 (0.73, 1.67)	0.64	9.59 (0.96, 95.78)	0.05
Education				
Less than high school	ref	ref	ref	ref
High school or equivalent	0.81 (0.66, 0.98)	0.03	2.62 (1.18, 5.83)	0.02
Some college or AA degree	0.70 (0.58, 0.86)	<0.001	2.69 (1.06, 6.79)	0.04
College graduate or above	0.56 (0.46, 0.70)	<0.0001	1.55 (0.50, 4.77)	0.45
Marital status				
Married	ref	ref	ref	ref
Never married	1.65 (1.30, 2.09)	<0.0001	5.05 (2.02, 12.59)	<0.001
Living with partner	1.47 (1.07, 2.03)	0.02	12.59 (3.88, 40.86)	<0.0001
Other	1.73 (1.50, 2.00)	<0.0001	2.13 (1.05, 4.34)	0.04
BMI category				
<25	ref	ref	ref	ref
25–30	0.77 (0.67, 0.89)	<0.001	0.88 (0.39, 1.97)	0.75
≥30	0.79 (0.68, 0.90)	<0.001	1.43 (0.73, 2.80)	0.29
Smoking status				
Never	ref	ref	ref	ref
Former	1.19 (1.00, 1.42)	0.04	1.46 (0.55, 3.85)	0.45
Now	2.00 (1.71, 2.33)	<0.0001	2.85 (0.89, 9.18)	0.08
Drinking status				
Never	ref	ref	ref	ref
Former	1.30 (1.04, 1.61)	0.02	3.49 (0.93, 13.08)	0.06
Now	0.76 (0.61, 0.94)	0.01	1.48 (0.42, 5.22)	0.54
Diabetes				
No	ref	ref	ref	ref
IGT	1.21 (0.92, 1.60)	0.18	1.30 (0.31, 5.50)	0.72
IFG	1.30 (0.97, 1.76)	0.08	3.24 (1.13, 9.36)	0.03
DM	1.71 (1.47, 2.00)	<0.0001	2.26 (1.04, 4.94)	0.04

BMI, body mass index; CI, confidence interval; DM, diabetes mellitus; HR, hazard ratio; IFG, impaired fasting glycaemia; IGT, impaired glucose tolerance.

## Discussion

In the current investigation, our objective was to unravel the intricate connections among psoriasis, hypertension, and mortality through a meticulous analysis of data derived from the NHANES spanning the periods 2003–2006 and 2009–2014. This study marks a pioneering effort, representing the first exploration into the collective impact of psoriasis and hypertension on mortality. Our findings illuminate a substantial elevation in the risk of all-cause mortality among individuals simultaneously grappling with psoriasis and hypertension, a phenomenon that persists even following rigorous adjustments for demographic factors (sex, age, race, education, marital status), BMI category, smoking status, drinking status, and diabetes. These results not only underscore the exigency of targeted psoriasis screening within the hypertensive demographic but also lay a robust foundation for further exploration in both foundational and clinical research.

Psoriasis, recognized as a chronic and recurrent systemic inflammatory disorder, is commonly accompanied by an array of comorbidities alongside its distinctive skin lesions. Conti A and colleagues found an association between microalbuminuria and the duration of psoriasis, as well as with psoriatic arthritis [15]. The investigation by Liu and colleagues demonstrated an increased susceptibility to thyroid disease among individuals with psoriasis in the United States [16]. Furthermore, a cross-sectional examination involving the adult outpatient population in the United States identified a correlation between psoriasis and nonalcoholic fatty liver disease [17]. Within the spectrum of comorbidities correlated with psoriasis, metabolic syndrome emerges as the predominant one, serving as an independent risk factor for cardiovascular disease—an eminent cause of mortality among individuals affected by psoriasis [18]. Duan et al. systematically reviewed and conducted a meta-analysis to quantify the association between psoriasis and hypertension. Their findings underscored an increased risk of hypertension in individuals with psoriasis compared to those without the condition [11]. Therefore, biological treatments targeting the underlying mechanisms of psoriasis can potentially alleviate the inflammatory burden associated with the condition and reduce the risk of developing various comorbidities.

Our investigation extends the groundwork laid by preceding longitudinal studies that have delved into the association between psoriasis and mortality. Gelfand et al. reported that the heightened risk of death was associated with severe psoriasis, but not mild psoriasis [19]. In a population-based cohort study in the UK, it was disclosed that despite an overall decrease in all-cause mortality rates over a 15-year span for both the general populace and individuals with psoriasis, the risk of all-cause mortality persists at an elevated level for psoriasis patients in comparison to those devoid of the condition [20]. Remarkably, two distinct meta-analyses have independently affirmed the association between psoriasis and an elevated risk of mortality [11, 21]. This association is, in part, explicable by the heightened prevalence of cardiovascular, infectious, and neoplastic disorders within the demographic of individuals with psoriasis [13]. A recent investigation systematically explored the joint influence of psoriasis and chronic kidney disease on mortality in a representative sample of U.S. adults, revealing a robust association between psoriasis and heightened all-cause mortality in individuals afflicted by chronic kidney disease [22]. Given the widespread prevalence of hypertension in the general population [1–3], the collective impact of psoriasis and hypertension on mortality risk remains an unresolved query.

This study included 19,799 individuals, among whom 554 had psoriasis and 7,692 had hypertension. The all-cause mortality of the population with psoriasis was higher compared to those without psoriasis, but the difference did not reach statistical significance. On the other hand, the all-cause mortality of individuals with hypertension was significantly higher compared to those without hypertension. In pursuit of a more comprehensive understanding of the collective influence of psoriasis and hypertension on mortality, we stratified all patients into four distinct groups. The results revealed that hypertension increased mortality compared to individuals without hypertension or psoriasis, while psoriasis alone did not significantly increase mortality. However, when psoriasis was present alongside hypertension, there was a significant increase in mortality, which was also higher compared to patients with hypertension alone. A plausible rationale for these findings lies in our choice of participants without a history of either psoriasis or hypertension as the reference group, implying that hypertension continues to exert a considerable influence on the attributable risk of mortality.

A significant observation in our study underscores the diminished survival evident in individuals concurrently affected by psoriasis and hypertension. Additionally, upon dividing patients into psoriasis and non-psoriasis populations, we observed that among the non-psoriasis population, the mortality risk was markedly elevated in participants with hypertension compared with those without hypertension. Among people with psoriasis, the mortality rate from hypertension was even higher. The mortality risk observed in this study was partially independent of psoriasis. Nonetheless, it is crucial to emphasize that the existence of psoriasis can serve as a substantial supplementary risk factor for all-cause mortality in the context of hypertension. These results bear significant implications, underscoring the potential critical role of screening for psoriasis in augmenting the life expectancy of individuals with hypertension. The mechanisms underpinning the interconnections between psoriasis, hypertension, and mortality remain indistinct. Psoriasis, characterized by chronic inflammation, is associated with a spectrum of systemic disorders, including plaques, psoriatic arthritis, and metabolic syndrome. There may be various factors contributing to the development of metabolic syndrome in these psoriasis patients, including chronic steroid usage. Various components of metabolic syndrome, such as obesity, diabetes, dyslipidemia, nonalcoholic fatty liver disease, and cardiovascular disease, are commonly identified in individuals affected by psoriasis [9]. The presence of multiple comorbidities in patients with psoriasis may contribute to an elevated risk of all-cause mortality.

As the most extensive investigation to date scrutinizing the synergistic collective influence of psoriasis and hypertension on mortality risk, this study boasts several salient strengths. Firstly, our analysis utilized nationally representative survey data with a long follow-up period. Furthermore, we applied appropriate statistical procedures and sample weights to obtain all-cause mortality that are reflective of the general population. The robust sample size of NHANES allowed for adjustment for potential confounding variables. Secondly, our study employed a random sampling approach to select participants from the general population, thus assuring the representativeness of our findings. Thus, our results can be extrapolated to the broader US population with confidence. Thirdly, as previously underscored, despite earlier investigations into the relationship between psoriasis and hypertension, our study uniquely stands as the pioneer in examining the conjoined impact of both conditions on mortality.

Our findings must be interpreted in light of certain constraints. Firstly, the NHANES data’s cross-sectional design precludes us from establishing causality between psoriasis, hypertension and all-cause mortality. Secondly, there might be sampling error due to cluster sampling method. Thirdly, despite our efforts to adjust for relevant confounding variables, there may still be residual confounding from unmeasured or unknown factors. Fourthly, only 276 participants had both psoriasis and hypertension. This population size was rather small to draw any final conclusion. Fifthly, the absence of detailed information regarding the severity of psoriasis in the NHANES data poses a limitation on our capacity to discern the relationship between all-cause mortality and distinct severity categories of psoriasis. Despite these constraints, our study revealed a positive association between psoriasis, hypertension and all-cause mortality. The implementation of screening measures and proactive management strategies for psoriasis and hypertension could potentially serve as an effective approach to reduce mortality risk, with potential implications for both clinical practice and public health. Future research with larger sample sizes and more precise measurement methods is essential to determine whether combination strategies could improve prognosis of patients with both psoriasis and hypertension.

## Conclusions

Our study unveiled a notable and positive association between psoriasis, hypertension, and all-cause mortality, underscoring an increased mortality risk for individuals concurrently affected by both conditions. There is a pressing need for additional research to gain a more profound understanding of the intricate relationships existing among psoriasis, hypertension, and all-cause mortality, and to scrutinize the potential therapeutic benefits specific to this particular population.

## Supporting information

S1 Table Association between demographic and clinical characteristics of the participants and all-cause mortality. (DOCX)

S1 Data (XLSX)
