# Supplementary material for: Synergistic impact of psoriasis and hypertension on all-cause mortality risk: A prospective cohort study
Source: PLoS One. 2024 Jul 5;19(7):e0306048. doi: 10.1371/journal.pone.0306048 (PMC11226118; doi:10.1371/journal.pone.0306048)
Supplement: S1 Table — (DOCX) [file pone.0306048.s001.docx]

**S1 Table** Association between demographic and clinical characteristics of the participants and all-cause mortality.

| Variable | | Multivariable model | | | |
| --- | --- | --- | --- | --- | --- |
|  |  | HR (95% CI) | | *P*-value | |
| Psoriasis | |  | |  | |
| No | | ref | | ref | |
| Yes | | 1.17 (0.84, 1.62) | | 0.36 | |
| Hypertension | |  | |  | |
| No | | ref | | ref | |
| Yes | | 1.81 (1.58, 2.07) | | <0.0001 | |
| Sex | |  | |  | |
| Female | | ref | | ref | |
| Male | | 1.41 (1.28, 1.55) | | <0.0001 | |
| Age group | |  | |  | |
| ≤49 | | ref | | ref | |
| 50-65 | | 3.38 (2.71, 4.21) | | <0.0001 | |
| ≥65 | | 13.30 (10.55,16.79) | | <0.0001 | |
| Race | |  | |  | |
| Hispanic | | ref | | ref | |
| Non-Hispanic White | | 1.70 (1.27, 2.28) | | <0.001 | |
| Non-Hispanic Black | | 1.49 (1.11, 2.00) | | 0.01 | |
| Mexican American | | 1.12 (0.85, 1.49) | | 0.41 | |
| Other | | 1.16 (0.78, 1.72) | | 0.47 | |
| Education | |  | |  | |
| Less than high school | | ref | | ref | |
| High school or equivalent | | 0.84 (0.68, 1.03) | | 0.09 | |
| Some college or AA degree | | 0.73 (0.60, 0.90) | | 0.003 | |
| College graduate or above | | 0.59 (0.48, 0.72) | | <0.0001 | |
| Marital status | |  | |  | |
| Married | | ref | | ref | |
| Never married | | 1.70 (1.35, 2.14) | | <0.0001 | |
| Living with partner | | 1.56 (1.15, 2.12) | | 0.004 | |
| Other | | 1.73 (1.50, 2.00) | | <0.0001 | |
| BMI category | |  | |  | |
| <25 | | ref | | ref | |
| 25-30 | | 0.77 (0.67, 0.89) | | <0.001 | |
| ≥30 | | 0.81 (0.70, 0.93) | | 0.003 | |
| Smoking status | |  | |  | |
| Never | | ref | | ref | |
| Former | | 1.20 (1.02, 1.41) | | 0.03 | |
| Now | | 2.01 (1.74, 2.32) | | <0.0001 | |
| Drinking status | |  | |  | |
| Never | | ref | | ref | |
| Former | | 1.34 (1.09, 1.65) | | 0.01 | |
| Now | | 0.77 (0.62, 0.96) | | 0.02 | |
| Diabetes | |  | |  | |
| No | | ref | | ref | |
| IGT | | 1.21 (0.92, 1.60) | | 0.17 | |
| IFG | | 1.33 (1.00, 1.78) | | 0.05 | |
| DM | | 1.70 (1.46, 1.98) | | <0.0001 | |

BMI, body mass index; CI, confidence interval; DM, diabetes mellitus; HR, hazard ratio; IFG, impaired fasting glycaemia; IGT, impaired glucose tolerance.
